# Combined Influence of *EGF+61G>A* and *TGFB+869T>C* Functional Polymorphisms in Renal Cell Carcinoma Progression and Overall Survival: The Link to Plasma Circulating MiR-7 and MiR-221/222 Expression

**DOI:** 10.1371/journal.pone.0103258

**Published:** 2015-04-24

**Authors:** Ana L. Teixeira, Francisca Dias, Marta Ferreira, Mónica Gomes, Juliana I. Santos, Francisco Lobo, Joaquina Maurício, José Carlos Machado, Rui Medeiros

**Affiliations:** 1 Molecular Oncology Group & Virology Pathology, Portuguese Institute of Oncology of Porto, Porto, Portugal; 2 Abel Salazar Institute for the Biomedical Sciences (ICBAS) of University of Porto, Porto, Portugal; 3 Research Department, Portuguese League Against Cancer (NRNorte), Porto, Portugal; 4 Oncology Department, Portuguese Institute of Oncology of Porto, Porto, Portugal; 5 Urology Department, Portuguese Institute of Oncology of Porto, Porto, Portugal; 6 Institute of Molecular Pathology and Immunology of the University of Porto (IPATIMUP), Porto, Portugal; 7 Faculty of Medicine- University of Porto, Porto, Portugal; 8 Faculty of Health Sciences of Fernando Pessoa University, Porto, Portugal; Yale School of Public Health, UNITED STATES

## Abstract

The epidermal growth factor (EGF) is responsible for the activation of intracellular signal transducers that act on cell-cycle progression, cell motility, angiogenesis and inhibition of apoptosis. However, cells can block these effects activating opposite signaling pathways, such as the transforming growth factor beta 1 (TGFβ1) pathway. Thus changes in expression levels of *EGF* and *TGFB1* in renal cells might modulate the renal cell carcinoma (RCC) development, in consequence of changes in regulatory elements of signaling networks such as the microRNAs (miRNAs). Our purpose was to investigate the synergic role of *EGF+61G>A* and *TGFB1+869T>C* polymorphisms in RCC development. Genetic polymorphisms were studied by allelic discrimination using real-time PCR in 133 RCC patients *vs*. 443 healthy individuals. The circulating EGF/EGFR-MAPK-related miR-7, miR-221 and miR-222 expression was analyzed by a quantitative real-time PCR in plasma from 22 RCC patients vs. 27 healthy individuals. The intermediate/high genetic proliferation profile patients carriers present a significantly reduced time-to-progression and a higher risk of an early relapse compared with the low genetic proliferation profile carriers (HR = 8.8, *P* = 0.038) with impact in a lower overall survival (Log rank test, *P* = 0.047). The RCC patients presented higher circulating expression levels of miR-7 than healthy individuals (6.1-fold increase, *P*<0.001). Moreover, the intermediate/high genetic proliferation profile carriers present an increase in expression levels of miR-7, miR-221 and miR-222 during the RCC development and this increase is not observed in low genetic proliferation profile (*P*<0.001, *P* = 0.004, *P*<0.001, respectively). The stimulus to angiogenesis, cell-cycle progression and tumoral cells invasion, through activation of EGFR/MAPK signaling pathway in intermediate/high proliferation profile carriers is associated with an early disease progression, resulting in a poor overall survival. We also demonstrated that the intermediate/high proliferation profile is an unfavorable prognostic factor of RCC and miR-7, miR-221 and miR-222 expressions may be useful phenotype biomarkers of EGFR/MAPK activation.

## Introduction

Renal cell carcinoma (RCC) is responsible for approximately 3% of all cancers in adult, with incidence rates increasing 2% per year [1,2]. RCC is the most common solid cancer of the adult kidney and comprises different tumour types with different molecular mechanisms leading to disease and, therefore, different treatment approaches. Unfortunately, one third of the patients present metastatic disease at diagnosis and 20–40% of RCC patient’s submitted to surgical nephrectomy will develop metastasis [3]. Currently, the increasing knowledge of the RCC molecular biology allows the development of new biologic therapies, with the purpose to stop cancer progression, blocking the angiogenesis and tyrosine kinase domains. Despite their promising role, resistance developed after a median of 5–11 months [4,5,6].

An aberrant activation of oncogenic signaling pathways, including the up-regulation of the pathway activated by the epidermal growth factor (EGF) and the loss of tumor suppressor pathways, such as the down-regulation of the transforming growth factor beta 1 (TGFβ1) signaling pathway, have been characterized as hallmarks of cancer development and progression [7,8]. Cell homeostasis is regulated by the concerted action of both mitogenic growth and anti-proliferative signals that converge on regulators of the cell cycle. Changes in expression levels of EGF and the TGFβ1 can disrupt this homeostasis and promote the cancer progression.

EGF activates several pro-oncogenic intracellular pathways leading to tumor cell proliferation, cell cycle progression, angiogenesis and inhibition of apoptosis [8]. In normal conditions, a precise control of this pathway is imperative due to its involvement in renal organogenesis and electrolyte homeostasis [9]. However, it has been reported, an increase of 50–90% in epidermal growth factor receptor (EGFR) expression in RCC, being this up-regulation associated with higher cancer grade and worse prognosis [10]. Moreover, it has been proposed that the inactivation of the von Hippel Lindau factor (*VHL*) can increase the half-life of EGFR [11]. This higher stabilization of EGFR during RCC development can result in the stimulation of cell proliferation and apoptosis inhibition, favoring migration, invasion and tumor angiogenesis [8]. A functional genetic polymorphism in the *EGF* gene characterized by a G>A transition has been described in the 5’-untranslated region (rs4444903), and has been subject of investigation in several studies involving different types of cancer [12,13,14]. Moreover, functional studies showed that *EGF+61GG* carriers have an increased EGF production in both normal and tumoral cells [12,15].

The TGFβ1 is a multifunctional regulatory polypeptide that regulates mammalian development and differentiation, and has a key role in development and tissue homeostasis [16]. Paradoxically, it has been suggested to play a dual role during tumor development, acting as a tumor suppressor in the early stages and as a tumor promoter in the later stages [17]. In RCC the TGFβ1 expression levels can be correlated with tumor stage, being the TGFβ1 levels significantly elevated in RCC patients with metastatic disease [18,19]. A functional polymorphism was described in *TGFB1* gene, responsible for a T-to-C substitution at nucleotide 29 of codon 10 (rs1982073). This variant is located in the hydrophobic core of the signal peptide, resulting in the replacement of a hydrophobic leucine with a small, neutral proline, being this transition associated with higher circulating levels of TGFβ1 [20].

Functional genetic polymorphisms influencing EGF and TGFβ1 levels can induce changes in cellular microenvironment, which may disrupt renal homeostasis and contribute to RCC development and progression. Furthermore, recently it has been proposed that changes in growth factors levels can modulate the activation of cellular signaling pathways, influencing the expression of specific messenger RNA and microRNAs (miRNAs) [21,22]. MiRNAs are a family of small non-coding RNAs (19–25 nucleotides in length) that regulates gene expression by sequence-selective targeting of mRNAs, leading to degradation or blockade of mRNA at the post-transcriptional level [23]. They can be defined as key regulators in many biological processes including cell development, differentiation, apoptosis and proliferation[23]. An increase in EGF bioavailability, in consequence of the *EGF+61G>A* polymorphism, can induce a higher activation of the EGFR-RAS-RAF-MEK pathway, which can affect the expression of miRNAs involved in cell proliferation control, angiogenesis, invasion and metastasis formation. The miR-7 and miR-221/222 have been identifying as downstream transcriptional targets of the EGFR-RAS-RAF-MEK pathway [24,25]. Recently, Yu and co-workers described for the first time the potential oncogenic role of miR-7 in RCC cells [26]. Furthermore we also observed that RCC patients present higher expression levels of miR-221/222 than healthy individuals, being this increase associated with a lower patients’ overall survival [27].

Our purpose was to investigate the combined effect of *EGF+61G>A* and *TGFB1+869T>C* functional polymorphisms in RCC development and progression. The circulating levels of miR-7 and miR-221/222 will be used as phenotype biomarkers for EGFR signaling pathway activation, since these are EGFR-MAPK-related miRNAs [28].

## Material and Methods

### Ethics statement

The study was conducted according to the principles of the Helsinki Declaration. The study was approved by the local ethics committee at the Portuguese Institute of Oncology of Porto (Portugal). All individuals signed a written informed consent to participate in the study.

### Study population

One hundred and thirty-three patients (age 61.8±11.6 years) with histopathologically confirmed RCC were recruited at the Portuguese Institute of Oncology of Porto, from January 1999 to March 2009 (67.2% males and 32.3% females). In cases the extension of disease were classified according to TNM classification system of the American Joint Committee on Cancer (AJCC) 2010, 7^a^ edition (Table 1). Disease progression was defined as the period between the six and the thirty-six months after the date from nephrectomy to the date that local recurrence (8.6% cases) or metastasis was detected (91.4% cases) (mainly in lung, bone and liver or combined), the median follow-up time was 23 months (range: 7–36 months) [29,30].

**Table 1:** Population characteristics.

	Cases (%)	Control (%)
**Gender**		
Male	90 (67.7)	294 (66.4)
Female	43 (32.3)	159 (33.6)
**Age (years)** *	61.8±11.6	51.7±11.6
**Histology**		
Clear cells	95 (71.4)	
Papillary	13 (9.8)	
Chomophobe	19 (14.3)	
others	6 (4.5)	
**Clinical stage**		
I-II	90 (67.7)	
III-IV	43 (32.3)	
**T**		
T1	71 (53.4)	
T2	21 (15.8)	
T3	41 (30.8)	
**N**		
N0-N2	11 (8.3)	
Nx	122 (91.7)	
**M**		
M0	126 (94.7)	
M1	7 (5.3)	
**Tumor size**		
< 4 cm	34 (25.6)	
≥ 4 cm	99 (74.4)	
**Fuhrman grade**		
G1-G2	60 (45.1)	
G3-G4	71 (53.4)	
Gx	2 (1.5)	
**Microvascular invasion**		
Yes	114 (85.7)	
No	9 (6.8)	
Unknown	10 (7.5)	
**Leibovich score**		
Low risk	37 (39.0)	
Intermediate risk	31 (32.6)	
High risk	19 (20.0)	
Unknown	8 (8.4)	

*Mean ± standard deviation

Subjects without known history of cancer were recruited from the Portuguese Institute of Oncology of Porto Centre blood donor’s bank (age 51.7±11.6 years) and included in the control group (n = 443, 66.4% males and 33.6% females) (Table 1). Peripheral venous blood samples were collected from each subject enrolled in the study. Patient’s blood samples were obtained before the surgery.

### *EGF+61G>A* (rs4444903) and *TGFB1 +869T>C* (rs1982073) polymorphisms genotyping

After DNA extraction using the QIAamp DNA Mini kit (Qiagen) according to the manufacturer’s protocol, the polymorphisms were analyzed by allelic discrimination using 7300 real-time PCR System (Applied Biosystems). The specific reactions were based on a 5' nuclease PCR assay, using a TaqMan assay, which includes two allele-specific TaqManMGB probes (Applied Biosystems) containing distinct fluorescent dyes and a PCR primer pair to detect the specifics single nucleotide polymorphisms (SNPs). Real-time PCR was carried out using a 6 μL reaction mixture, containing 1x master mix (Applied Biosystems), with 1x probes (TaqManassays, C__27031637_10, C__22272997_10, respectively, Applied Biosystems) and 20 ng of the DNA sample. Thermal conditions were 95°C during 10 minutes for DNA polymerase activation, followed by 45 PCR cycles at 92°C for 15 seconds and 60°C for 1 minute. Quality control procedures implemented for genotype analyses included double sampling in 10% of the samples to assess reliability and the use of negative controls to step-away false positives. The ambiguous results were reanalyzed.

### Circulating plasma miRNA relative quantification

The miRNA-7 and miR-221/222 expression levels were analyzed by a quantitative real-time PCR, using plasma samples. After genotyping, 49 individuals were randomly chosen among the patients (n = 22) and the healthy individuals (n = 27) and plasma miRNAs were isolated using the commercial kit *GRS microRNA Kit* (GRISP) according to the manufacturer’s instructions. MicroRNA samples were then used as a template for cDNA synthesis using *TaqManMicroRNA Reverse Transcription Kit* (Applied Biosystems). The circulating miRNAs expression levels were analysed by a quantitative real-time PCR. Reactions were carried out on a StepOne One qPCR machine, containing 1x Master Mix (Applied Biosystems), with 1x probes (TaqMan MicroRNA Assays miR-7: 002314, miR221: 002096, miR-222: 002097, Applied Biosystems), cDNA sample, and the RNU48 endogenous control (TaqMan MicroRNA Assay: 001006, Applied Biosystems) was used to normalize the results, regarding the two biomarkers, since it presents a constant expression level. The data analysis was carried out using the *StepOne Software v2*.*2* (Applied Biosystems) with the same baseline and threshold set for each plate, in order to generate threshold cycle (*Ct*) values for all the genes in each sample.

### Statistical analysis

Data analysis was performed by the computer software IBM SPSS Statistics for Windows (Version 20.0). Genotypes of the two polymorphisms analyzed were combined into three levels considering the functional consequence of the polymorphisms in the modulation of cell proliferation [12,15,20]: high- (*EGF+61AG/GG and TGFB1+869TT)*, low- (*EGF+61AA* and *TGFB1+869CT/CC*) and intermediate- genetic proliferation profile (EGF+61AG/GG and *TGFB1+869CT/CC or EGF+61AA TGFB1+869TT*). The rationale for defining high genetic proliferation profile was to combine the G-allele from *EGF+61G>A* polymorphism, associated with higher expression levels of *EGF* with the TT genotype from *TGFB1+869T>C* variant related to lower TGFβ1 production. In low genetic proliferation profile we associated the lower-expressing AA genotype of *EGF+61G>A* polymorphism with *TGFB1+869TC/CC* variants, associated with higher circulating levels of TGFβ1. The Hardy–Weinberg equilibrium was tested by a Pearson chi-square analysis to compare the observed versus the expected genotype frequencies. The odds ratio (OR) and its 95% confidence interval (95% CI) were calculated as a measurement of the association between functional profiles and the RCC risk. A Cox proportional hazard model was used to analyze the time to progression defined as the period between the six and the thirty-six months after the nephrectomy, considering as covariates, age, gender, Leibovich score at diagnosis. Cox regression models were used to adjust for potential confounder. The Kaplan-Meier method and log-rank test were used to compare proliferation profiles influence in the overall survival. The 2^-ΔΔCt^ method was used to evaluate fold change in normalized expression of each miRNA as previously described [31]. Comparison of miRNAs expression levels between the genetic profiles were performed using Student’s t-test, in order to evaluate any statistical differences in the normalized expression of the miR-7, miR-221 and miR-222 explored.

## Results

Frequencies for homozygous AA and AG/GG genotypes of *EGF+61G>A* polymorphism were, respectively, 0.38 and 0.62 for RCC patients and 0.35 and 0.65 in the control group. The *TGFB1+869T>C* polymorphism frequencies for homozygous TT and CC/CT genotypes carriers were 0.36 and 0.64 in RCC group and 0.33 and 0.67 in the control group, respectively. Observed *versus* expected genotype frequencies were calculated and no deviation from Hardy–Weinberg equilibrium was observed, except for the *TGFB1+869T>C* polymorphism in patients group (*EGF+61G>A*: RCC group, *P* = 0.073, control group, *P* = 0.078; *TGFB1+869T>C*: RCC group, *P* = 0.023, control group, *P* = 0.195).

The genetic proliferation profiles distribution in cases and controls are present in Table 2. The present results show no statistically significant association among the profiles and the risk for RCC development (OR = 1.01, 95% CI: 0.62–1.65, *P* = 0.971; OR = 1.12, 95% CI: 0.63–2.00, *P* = 0.698).

**Table 2:** Genetic proliferation profile-related odds ratio for RCC and genotype frequencies in patients and control.

	Control Group	RCC Group	OR	95% CI	*P*
Proliferation Profiles					
Low	103 (0.23)	30 (0.23)	Referent		
Intermediate	245 (0.55)	72 (0.54)	1.01	0.62–1.65	0.971
High	95 (0.22)	31 (0.23)	1.12	0.63–2.00	0.698

RCC, renal cell carcinoma; OR, odds ratio; 95% CI, 95% confidence interval

Additionally, although the association was not statistically significant, there was a trend to an overrepresentation of the intermediate/high genetic proliferation profile in the patient group that present cancer progression compared with the individuals without cancer progression (OR = 6.08, 95% CI: 0.77–47.68, *P* = 0.083). Although, we did not observed any statistical association between the genetic proliferation profiles and cancer stage (OR = 0.94, 95% CI: 0.40–2.32, *P* = 0.893), Fuhrman grade (OR = 0.88, 95% CI: 0.38–2.01, *P* = 0.757) or presence of metastasis on diagnosis (OR = 1.79, 95% CI: 0.25–42.9, *P* = 0.590) (Table 3).

**Table 3:** RCC phenotype disease according genetic proliferation profiles.

Proliferation Profiles	Fuhrman Grade				
	G1-G2	G3-G4	OR	95% CI	*P*
Low	13 (0.22)	17 (0.24)	Referent		
Intermediate/high	47 (0.78)	54 (0.76)	0.88	0.38–2.01	0.757
	**Clinical Stage**				
	**I-II**	**III-IV**	**OR**	**95% CI**	***P***
Low	20 (0.22)	10 (0.23)	Referent		
Intermediate/high	70 (0.78)	33 (0.77)	0.94	0.40–2.32	0.893
	**TNM**				
	**M0**	**M1**	**OR**	**95% CI**	***P***
Low	29 (0.23)	1 (0.14)	Referent		
Intermediate/high	97 (0.77)	6 (0.86)	1.79	0.25–42.9	0.590
	**Leibovich score**				
	**Low risk**	**Intermediate/high risk**	**OR**	**95% CI**	***P***
Low	13 (0.27)	16 (0.21)	Referent		
Intermediate/high	36 (0.73)	60 (0.79)	1.35	0.57–3.16	0.479
	**Cancer Progression**				
	**No**	**Yes**	**OR**	**95% CI**	***P***
Low	27 (0.25)	1 (0.05)	Referent		
Intermediate/high	80 (0.75)	18 (0.95)	**6.08**	**0.77–47.68**	**0.083** *

OR, odds ratio; 95% CI, 95% confidence interval

* Fisher exact test

**Fig 1:**
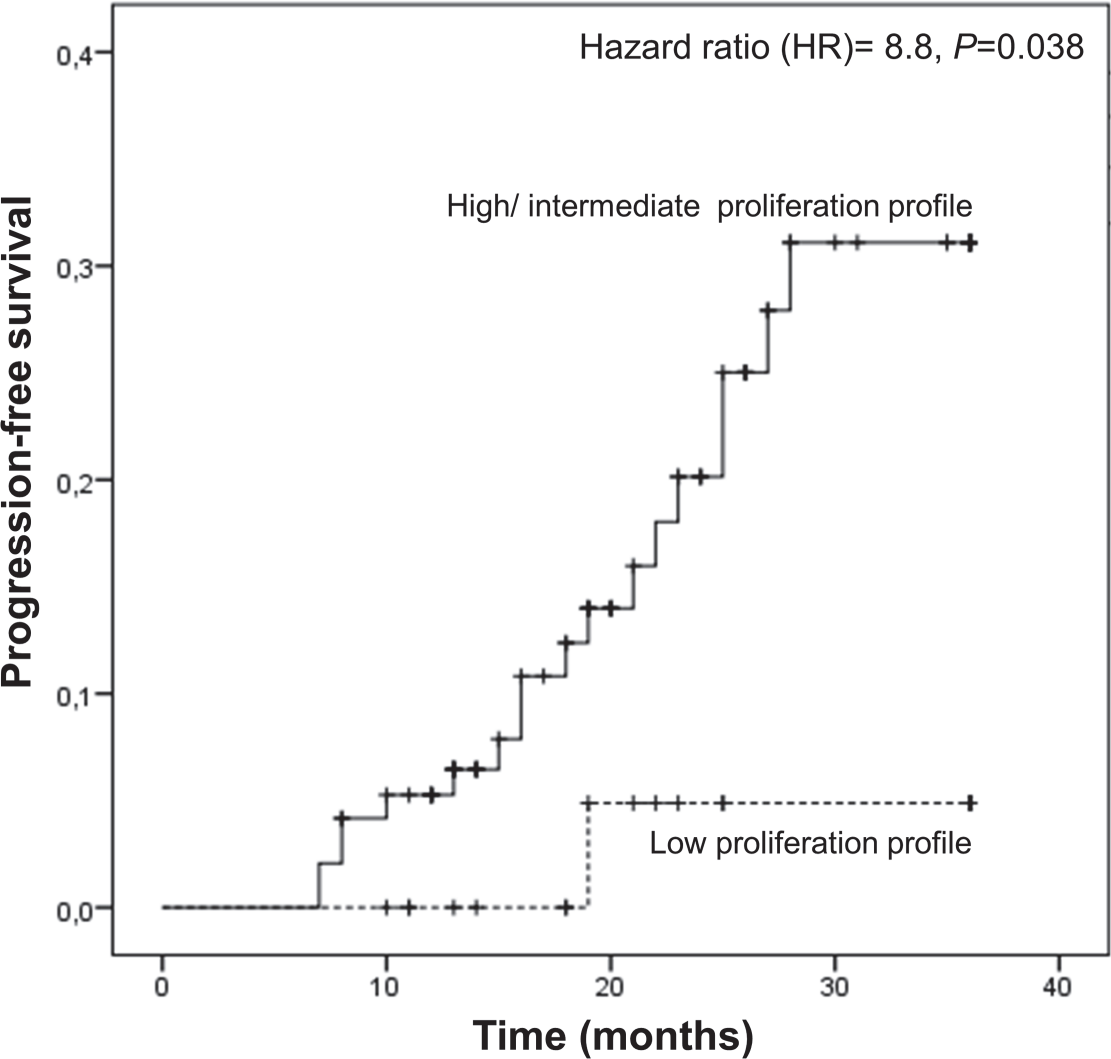
Time to disease progression according to genetic proliferation profiles in RCC patients. Hazard ratio using age, gender, Leibovich score at diagnosis as covariants.

Concerning the time to disease progression, we observed that the intermediate/high genetic proliferation profile carriers present a significantly reduced time-to-progression compared with the low genetic proliferation profile carriers (31.5 *vs* 35.2, Log Rank test, *P* = 0.043). Furthermore, multivariate Cox regression model using age, gender and Leibovich score as covariants, demonstrated a higher risk of earlier relapse in patients with intermediate/high proliferation profiles (hazard ratio- HR = 8.8, 95%CI: 1.13–68.24, *P* = 0.038) (Fig 1).

**Fig 2:**
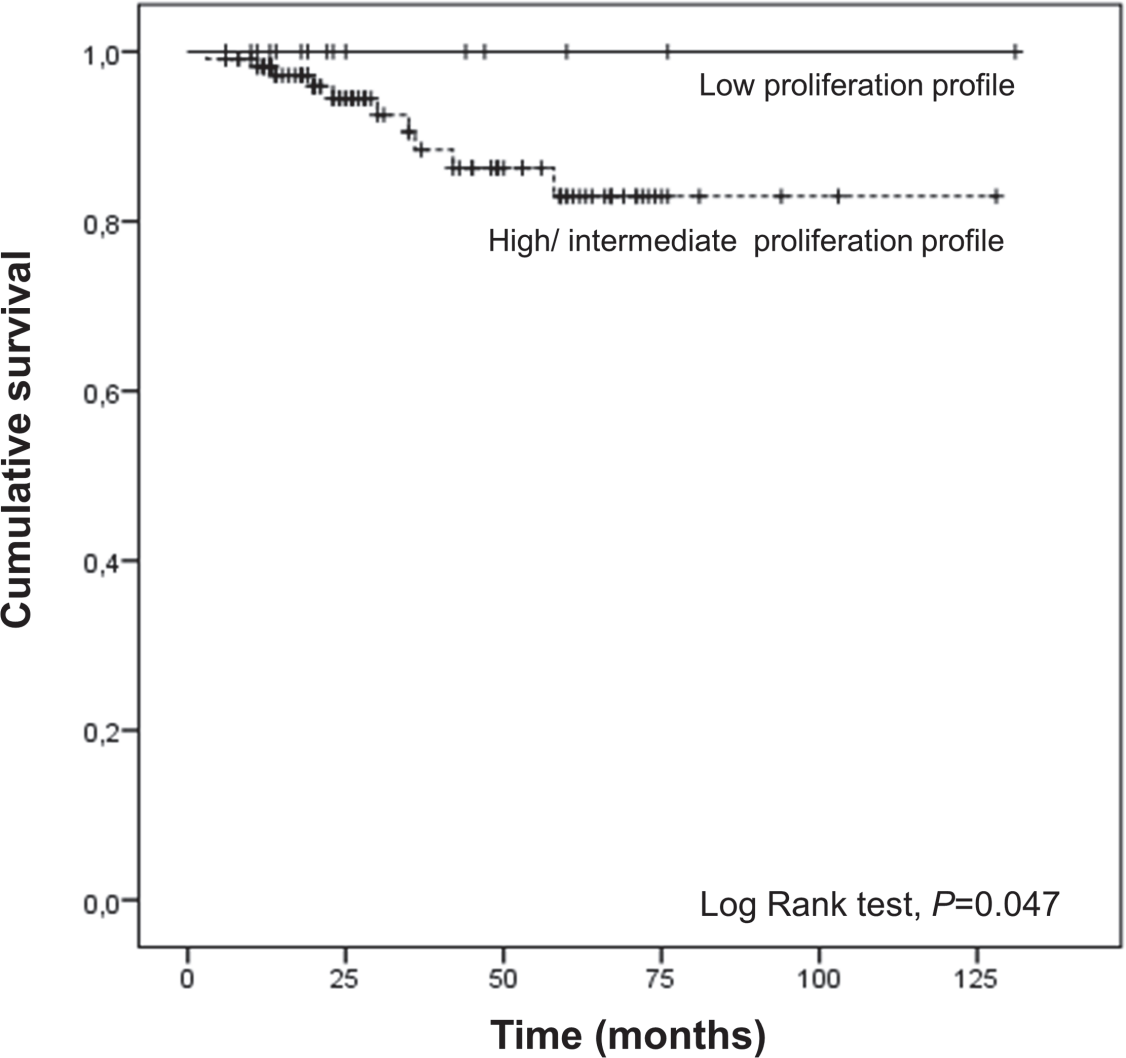
Overall survival of RCC patients according the genetic proliferation profile.

We also observed that RCC patients carriers of intermediate/high genetic proliferation profile have a lower overall survival than patients carriers of the low genetic proliferation profile (Log rank test, *P* = 0.047) (Fig 2).

**Fig 3:**
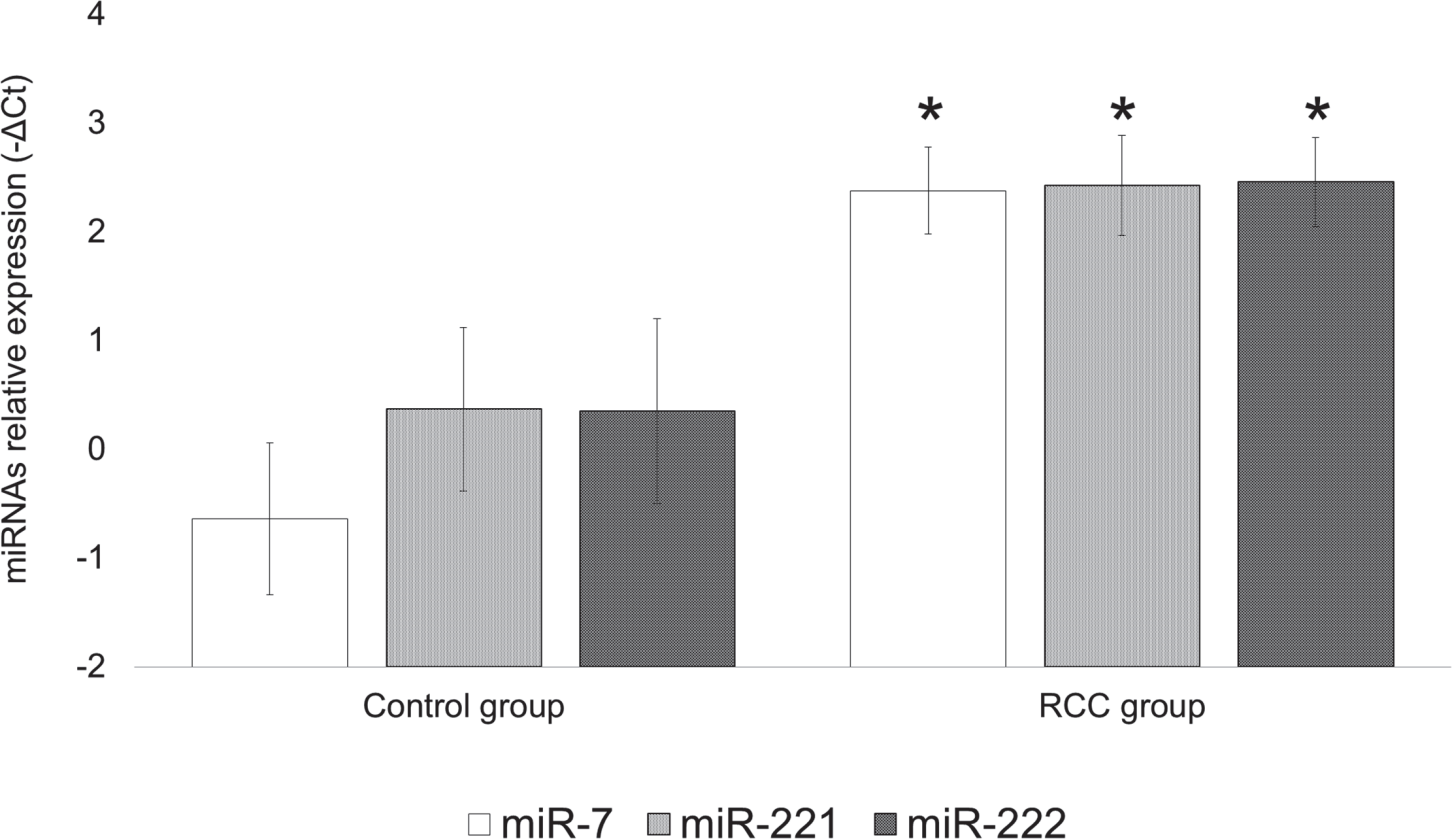
Plasma expression of miR-7, miR-221 and miR-222 in controls and RCC patients. Bars indicate mean± standard error of mean. *, *P*<0.050.

Regarding the circulating miRNAs expression, we detected changes in plasma expression levels of miR-7, miR-221 and miR-222 in our study population. We observed that RCC patients presented higher circulating expression levels of miR-7, miR-221 and miR-222 than healthy individuals (miR-7: 2^-ΔΔCt^ = 6.1, *P*<0.001; miR-221: 2^-ΔΔCt^ = 4.2, *P* = 0.035; miR-222: 2^-ΔΔCt^ = 4.3, *P* = 0.042) (Fig 3). However, we didn’t find statistical differences between miR-7, miR-221 and miR-222 expression levels according gender in control group (miR-7: *P* = 0.976; miR-221: *P* = 0.069; miR-222: *P* = 0.083) and in RCC group (miR-7: *P* = 0.087; miR-221: *P* = 0.534; miR-222: *P* = 0.795).

Considering, the tumor subtype in patient group we also didn’t find statistical differences in miR-7, miR-221 and miR-222 expression levels (miR-7: clear cell *vs* papillary *P* = 0.219, clear cell *vs* chomophobe *P* = 0.498, papillary *vs* chomophobe *P* = 0.086; miR-221: clear cell *vs* papillary *P* = 0.628, clear cell *vs* chomophobe *P* = 0.222, papillary *vs* chomophobe *P* = 0.588; miR-222: clear cell *vs* papillary *P* = 0.905, clear cell *vs* chomophobe *P* = 0.589, papillary *vs* chomophobe *P* = 0.678).

**Fig 4:**
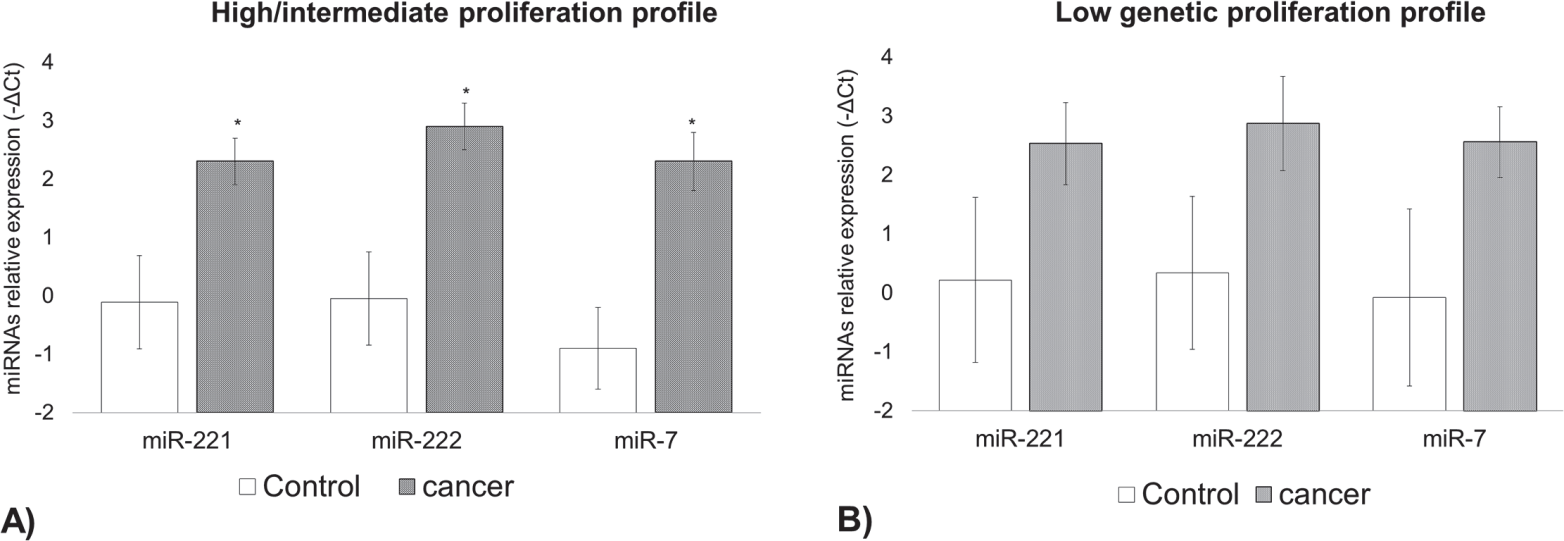
Plasma expression of miR-221, miR222 and miR-7 during the RCC development. Bars indicate mean± standard error of mean. *, *P*<0.050.

Interesting, when we compared the expression levels of miR-7, miR-221 and miR-222 in intermediate/high genetic proliferation profile carriers during the RCC development, we found an increase in expression levels of these miRNAs during the course of the disease (miR7: 2^-ΔΔCt^ = 9.2, *P*<0.001; miR-221: 2^-ΔΔCt^ = 5.3, *P* = 0.004; miR-222: 2^-ΔΔCt^ = 7.7, *P*<0.001) (Fig 4A)). However, we didn’t observed statistical differences in miRNAs expression in carriers of the low genetic proliferation profile during RCC development (Fig 4B)).

## Discussion

The RCC is a heterogeneous disease, with high potential to metastasize, and today’s therapeutic strategies are insufficient. The molecular heterogeneity of RCC reflects differences in disease course, tumor drug resistance, therapy effectiveness and prognosis. During RCC progression, the modulation of pro-oncogenic and pro-inhibitors factors ratios, could accelerate the tumor development and the acquisition of resistant phenotypes to anticancer therapies [5].

The EGF is a growth factor responsible for the activation of several intracellular signal transducers responsible for cell-cycle progression, cell motility, angiogenesis and inhibition of apoptosis, processes that must be up-regulated during carcinogenesis. On the other hand, in homeostatic conditions the cells can block these effects by activating opposite signaling pathways, such as the TGFβ1 pathway, involved in inhibition of cell proliferation, stopping the cell cycle and inducing the apoptosis [28].

We hypothesize that during RCC progression in cellular microenvironment the growth factors imbalance, in consequence of higher expression levels of mitogenic growth factors and lower levels of antiproliferative growth factors, such as high EGF or TGFα, and low levels of TGFβ1, could create a hiper-activation of the oncogenic EGF-TGFα/EGFR pathway, enhancing cancer progression and the acquisition of metastatic phenotypes. Our results suggest that changes in expression levels of EGF and TGFβ1 in consequence of functional single nucleotide polymorphisms (SNPs), may contribute to an homeostasis disequilibrium and thus to a higher risk for an earlier disease progression.

The tumor suppressor gene VHL is frequently lost in approximately 80% of all clear cell RCC (ccRCC), being this alterations a hallmark feature of this neoplasia, however additional events are required. This molecular event stops the degradation of HIF resulting in its accumulation in the cytoplasm and further migration to nucleus where it can activate the transcription of hypoxia related genes, such as TGFα, VEGF and PDGF. Zhou and co-workers describe that during the RCC development, the VHL inactivation can also lead to an increase of the EGFR [11]. Their study demonstrated that after EGF stimulation the phospho-AKT and the phospho-ERK signals lasted longer in 786-mock cells (VHL-/-/HIF1α-/-) than in 786-VHL cells (wild type) [11]. Previous studies have shown that up-regulation of EGFR is associated with high tumor grade and worse prognosis [32]. Recently, Brannon and co-workers proposed, that even within ccRCC occur different gene expression patterns, suggesting that the molecular profile could be responsible for different biological behaviors[33]. These authors distinguish two ccRCC subtypes, the ccA and ccB, considering the gene expression patterns and consequently the molecular pathways implicated in tumor development. The authors define a better prognosis group, the ccA group, associated with the overexpression of genes involved in hypoxia, angiogenesis, fatty acid and organic acid metabolisms. On the other hand, the ccB group was associated with the overexpression of more aggressive genes involved in cell differentiation, epithelial to mesenchymal transition, cell cycle, TGFβ pathway and wound healing, suggesting that these subgroup acquired additional genetic events that contribute to a more aggressive phenotype [33,34]. Brooks and co-workers also developed a biomarker signature which include the expression analysis of 34 genes, considering the two distinct ccRCC subtypes classification, good risk (ccA) and poor risk (ccB) [30]. Genetic and expression changes of several genes, that include TGFβ1, TGFβRII, EGFR, PTEN, AMPK among others, and their products interaction are involved in the ccRCC high complex molecular network [35,36].

The simultaneous deregulation of TGFβ1 signaling pathway is also implicated in renal carcinogenesis. This deregulation compromises the inhibition of cell cycle progression through G1-arrest, apoptosis, cyclin-dependent kinases inhibitors including p21^WAF1^ and p15^Ink4b^ and suppression of c-myc. The higher expression levels of TGFβ1 are observed in advanced disease. However, these higher circulating levels could be consequence of the impossibility of TGFβ1 binding to the type II receptor (TGFβRII). During tumor development, TGFβ1 binds to TGFβRII, initiating a signaling transduction that culminates in cell cycle control and induction of apoptosis. However, attenuation of this pathway could be partially explained by the loss of the TGFβRII. On the other hand, the restoration of this pathway is associated with an increase in the sensitivity of RCC cells to TGFβ1 [37]. Recently, we demonstrated that low expression levels of *TGFBR2* mRNA are associated with more aggressive prostate cancer phenotypes and with a higher risk to develop resistance to anticancer treatment [38].

It is evident that EGF and TGFβ1 signaling networks require a delicate balance of interactions within the cellular and tumoral microenvironment. Deregulation of *EGF* and *TGFB1* expression levels could influence the normal cellular homeostasis and also influence cancer progression. In the present study, we describe the combined effect of *EGF+61G>A* and *TGFB1+869T>C* polymorphisms, according to genetic proliferation profiles that could influence disease outcomes, such as the progression-free interval and the overall survival of RCC patients. Our results suggest that different bio-availability of growth factors, resulting from germline genetic variation, may modulate the tumoral microenvironment favoring the EGFR pathway activation. Moreover, EGF is one of the growth factors that lead to VEGF and MMP-9 expression, crucial mediators for tumor angiogenesis and invasion in the RCC microenvironment, therefore facilitating the spread of tumor cells [39,40,41].

The synergic lower TGFβ1 production associated with TT genotype and higher levels of EGF associated with the presence of G allele (intermediate/high genetic proliferation profile) might contribute to a favorable long term proliferative potential of RCC cells leading to a higher risk of disease progression. Accordingly, carriers of intermediate/high genetic proliferation constitutive profile will likely be exposed to an increased proliferative, angiogenic and invasive stimulus, thus contributing to an early development of metastatic disease, with impact in overall survival.

Functional studies demonstrated the functional consequence of the *EGF+61G>A* and *TGFB1+869T>C* polymorphisms. However, we demonstrated for the first time that the intermediate/high genetic proliferation profile patients’ carriers present higher miR-7 and miR-221/222 plasma circulating expression levels when compared with healthy individuals. This increase could be consequence not only due to the high expression levels of EGF but also an increase in the half-life of their receptor EGFR, induce by the loss of VHL during the RCC development. The simultaneous higher bioavailability of the preferential ligand EGF and a more stable EGFR could have as a consequence the stronger activation of the EGF/MAPK signaling, inducing higher expression of miR-7 and miR-221/222. We hypothesized, that the intermediate/high genetic proliferation profile carriers present a double disadvantage, the simultaneously increase of the EGFR and the higher EGF bioavailability, in consequence of their genetic background. Furthermore, the low genetic proliferation profile carriers present a trend to an increase expression level of these miRNAs, which suggest the combined influence of somatic alterations and the individual genetic background in the modulation of miR-7 and miR-221/222 plasma circulating expression levels. However, the small sample size in our study may limit the ability to discern meaningful differences. Further research is needed to evaluate the associations reported here in more details, as well as to evaluate the association of the proliferation genetic profile and the miRNAs expression levels with the two ccRCC subtypes, attending the dichotomous molecular mechanisms involved in the ccRCC development.

The miR-7, could be defined as a downstream effector of EGFR/MAPK pathway inducing the expression of cyclin A through inhibition of the repressor ERF and consequently the progression of the cell cycle. Studies performed by Chou and co-workers showed that the overexpression of miR-7 can stimulate cyclin A expression, suggesting that this miRNA can antagonize the ERF mediated cyclin A suppression [23]. Cumulatively, the miR-221/222 also have the ability to modulate cell cycle, repressing cell cycle inhibitor proteins p27/Kip1 and p57 inducing cell proliferation and self-renewal [42,43,44].

On the other hand, these three miRNAs seem to have the capacity to influence the epithelial-mesenquimal transition required for the metastatic process, inducing the expression of metalloproteinases (MMPs), such as the increase of MMP-2 and MMP-9. Recently Jung and co-workers demonstrated that miR-7 is involved in the regulation of the tumor suppressor gene reversion-inducing cysteine-rich protein with kazal motifs (RECK), causing its suppression [45]. The tumor suppressor RECK is able to suppress tumor angiogenesis, invasion and metastasis, inhibiting MMP-2 and MMP-9 [46]. The increase expression of miR-7 could inhibit the RECK mRNA, inducing the expression of MMP-2 and MMP-9. The deregulation of these two MMPs leads to an excessive degradative activity favoring the invasion of tumor cells [47]. The up-regulation of MMPs is associated with tumor progression [39]. Furthermore, Zhang and co-workers demonstrated that miR-221/222 knockdown decreased the invasion capability and tumor growth and up-regulated the expression of suppressor gene tissue inhibitor metallopeptidase 3 (TIMP3) [43,48].

In conclusion, the stimulus to angiogenesis, cell-cycle progression and invasion of tumoral cells, through activation of EGFR/MAPK signaling pathway in intermediate/high proliferation profile carriers is associated with an early disease progression, resulting in a poor overall survival of these patients. We also demonstrated that the intermediate/high proliferation profile is an unfavorable prognostic factor of RCC and miR-7 and miR-221/222 plasma expressions may be useful phenotype biomarkers of EGFR/MAPK activation during RCC development.
